# Dihydrotanshinone I Specifically Inhibits NLRP3 Inflammasome Activation and Protects Against Septic Shock *In Vivo*

**DOI:** 10.3389/fphar.2021.750815

**Published:** 2021-10-14

**Authors:** Ziying Wei, Xiaoyan Zhan, Kaixin Ding, Guang Xu, Wei Shi, Lutong Ren, Zhie Fang, Tingting Liu, Xiaorong Hou, Jia Zhao, Hui Li, Jiayi Li, Zhiyong Li, Qiang Li, Li Lin, Yan Yang, Xiaohe Xiao, Zhaofang Bai, Junling Cao

**Affiliations:** ^1^ School of Chinese Meteria Medica, Beijing University of Chinese Medicine, Beijing, China; ^2^ Senior Department of Hepatology, the Fifth Medical Center of PLA General Hospital, Bejjing, China; ^3^ China Military Institute of Chinese Materia, Fifth Medical Center of Chinese PLA General Hospital, Beijing, China; ^4^ Department of Pharmacy, Dongfang Hospital, Beijing University of Chinese Medicine, Beijing, China

**Keywords:** dihydrotanshinone I, NLRP3 inflammasome, caspase-1, IL-1β, septic shock

## Abstract

The abnormal activation of the NLRP3 inflammasome is closely related to the occurrence and development of many inflammatory diseases. Targeting the NLRP3 inflammasome has been considered an efficient therapy to treat infections. We found that dihydrotanshinone I (DHT) specifically blocked the canonical and non-canonical activation of the NLRP3 inflammasome. Nevertheless, DHT had no relation with the activation of AIM2 or the NLRC4 inflammasome. Further study demonstrated that DHT had no influences on potassium efflux, calcium flux, or the production of mitochondrial ROS. We also discovered that DHT suppressed ASC oligomerization induced by NLRP3 agonists, suggesting that DHT inhibited the assembly of the NLRP3 inflammasome. Importantly, DHT possessed a significant therapeutic effect on NLRP3 inflammasome–mediated sepsis in mice. Therefore, our results aimed to clarify DHT as a specific small-molecule inhibitor for the NLRP3 inflammasome and suggested that DHT can be used as a potential drug against NLRP3-mediated diseases.

## Introduction

An inflammasome is a cytoplasmic complex composed of multiple proteins, which mediates the host immune response to microbial infection and cell damage (Péladeau and Sandhu, 2021). The NLR family proteins are intracellular signaling molecules that can sense many factors of pathogenic origin, environment, and host origin (Su et al., 2021). Inflammasomes are composed of intracellular pattern recognition receptors (PRRs), including NLRP3, NLRP1, and NLRC4, belonging to the NLR family and melanoma 2 (AIM2) belonging to the non-NLR family. (Zhang WJ. et al., 2021). Among them, the study of the NLRP3 inflammasome is the most in-depth and extensive one.

Both pathogen-associated molecular patterns (PAMPs) and danger-associated molecular patterns (DAMPs) can activate inflammasomes. NLRP3, ASC (apoptosis-associated speck-like protein containing a card), and caspase-1 are three components of the NLRP3 inflammasome (Elliott and Sutterwala, 2015). NLRP3 binds to ASC, and ASC interacts with caspase-1, which triggers the self-cleavage of pro–caspase-1 to form mature caspase-1 and then leads to pyrosis and IL-1β production. The NLRP3 inflammasome could be activated by many types of pathogens or danger signals, such as nigericin, adenosine triphosphate (ATP), cholesterol crystal, amyloid-β aggregates, pore-forming toxins, or viral RNA (He et al., 2016a; Wang et al., 2019; Shi et al., 2020). In addition, the environmental factors, such as ultraviolet irradiation, SiO_2_, PM2.5, allergens, infectious agents, and physical stress, are also important for the activation of the NLRP3 inflammasome (Feldmeyer et al., 2007; Elliott and Sutterwala, 2015; Broz and Dixit, 2016; Chu et al., 2019). The NLRP3 inflammasome has been known to be a participant in the pathogenesis which is associated with many metabolic and inflammation-related diseases. Hereditary caps such as Muckle–Wells syndrome (MWS), neonatal multi-system inflammatory diseases, and familial cold auto-inflammatory syndrome result from functional acquisition mutations of the NLRP3 inflammasome (Awad et al., 2019; Iida et al., 2019; Nair et al., 2019). Moreover, NLRP3 inflammasome activation is conducive to the evolution of many other medical conditions, including Parkinson's disease (Han et al., 2019), inflammatory bowel disease (Chen et al., 2021), Alzheimer’s disease (Feng et al., 2020), Crohn’s disease (Zhang G. et al., 2021), and liver disease (Yang et al., 2020). Pharmacological inhibitors of the NLRP3 inflammasome have exhibited significant therapeutic efficacy in multiple animal models (Daniels et al., 2016; Wu et al., 2017; Renaudin et al., 2020). Therefore, the NLRP3 inflammasome is widely considered a new target for the treatment of inflammatory diseases. MCC950 (Corcoran et al., 2021), Olt1177 (Marchetti et al., 2018), cryptotanshinone (Liu et al., 2021), isoliquiritigenin (Honda et al., 2014), and tranilast (Zhuang et al., 2020) have been affirmed to repress the NLRP3 inflammasome over the years. MCC950 has been proven to be the most effective and specific inhibitor for NLRP3 inflammasome activation. It is effective in many NLRP3-driven diseased mouse models, such as Alzheimer’s disease (Tapia-Abellán et al., 2019), colitis, and type 2 diabetes. However, phase II clinical trials have demonstrated their potential hepatotoxicity (Shi et al., 2020; Corcoran et al., 2021). In addition, OLT1177 also conducted phase II clinical trials (Marchetti et al., 2018). However, there is no therapy targeting NLRP3 available in the clinic. It is essential to undertake an urgent study to develop secure and effective inhibitors on the NLRP3 inflammasome against NLRP3 inflammasome–mediated diseases.

*Salvia miltiorrhiza* Bge. (Lamiaceae) is one of the most famous traditional Chinese medicines. As a traditional Chinese medicine, Danshen is widely used to treat blood abnormalities, heart disease, hepatitis, bleeding, menstrual irregularities, collagen-induced platelet aggregation, and so on (Mei et al., 2019; Guo et al., 2021). Dihydrotanshinone I (DHT) is a natural compound extracted from Danshen. According to the reports, DHT has antitumor (Allegri et al., 2021), anti-inflammatory (Wang et al., 2020), and immunomodulatory effects (Wang et al., 2020). Evidence has emerged that DHT attenuates crystalline silica–induced lung inflammation by regulating the immune response and inhibiting STAT1/STAT3 (Wang et al., 2020). It has been reported that DHT could inhibit the activation of NF-κB induced by TNF-α (Weng et al., 2021). In this study, we discovered that DHT blocked the activation of the NLRP3 inflammasome but not NLRC4 and AIM2 inflammasomes. In addition to this, DHT treatment can protect against NLRP3 inflammasome–mediated infectious shock and inflammation *in vivo*, which indicates that DHT may have a potential as a therapeutic drug for the treatment of NLRP3 inflammasome–mediated diseases.

## Materials and Methods

### Mice

C57BL/6 female mice (6- to 8-week-old) were purchased from SPF Biotechnology Co., Ltd. (Beijing, China). Mice were kept under pathogen-free conditions. All mice were given unlimited water and food, and maintained under a 12-h dark/light cycle (25 ± 2°C). The animal experiments were carried out in consistence with the guidelines for the care, as well as use of laboratory animals and were approved by the Fifth Medical Center of PLA General Hospital, Beijing, China.

### Reagents and Antibodies

ATP, nigericin, and DMSO were obtained from Sigma. SiO_2_, Pam3CSK4, poly (dA:dT), and ultrapure lipopolysaccharide (LPS) were provided by InvivoGen. Dihydrotanshinone I (DHT) was purchased from MCE and Targetmol. *Salmonella* was supplied as a gift from Dr. Tao Li from the National Center of Biomedical Analysis (Beijing, China). MitoSOX was supplied by Invitrogen. Anti-mouse caspase-1 (1:1,000, AG-20B-0042) and anti-NLRP3 (1:2000, AG-20B-0014) were supplied from AdipoGen. Anti-ASC (1:1,000, #67824) and anti-GAPDH (1:1,000, #5174) were supplied by Cell Signaling Technology. Anti-mouse IL-1β (1:1,000, AF-401-SP) was obtained from R&D Systems. Anti-flag (1:1,000, 80010-1-RR), anti-NEK7 (1:1,000), and anti-lamin B (1:1,000, 10895-1-AP) were supplied by Proteintech. F4/80 (565,410) was obtained from BD Biosciences.

### Cell Culture

Bone marrow–derived macrophages (BMDMs) were derived from 10-week-old C57BL/6 female mice and cultured in Dulbecco’s modified Eagle’s medium (DMEM) containing 10% fetal bovine serum (FBS), 1% penicillin/streptomycin (P/S), and 50 ng/ml murine macrophage colony-stimulating factor (M-CSF, 416-ML-050, R&D Systems) for 6–7 days. HEK-293T was cultured in DMEM containing 10% FBS and 1% penicillin/streptomycin (P/S). All cells were cultured at 37°C humidification (5% CO_2_).

### Cell Viability Assay

BMDMs were seeded in 96-well plates overnight at a density of 1 × 10^5^ cells/well. Then, BMDMs were exposed to DHT in DMEM for 24 h at 37°C. Next, the medium was replaced with fresh DMEM containing CCK-8 for 15–30 min. The optical density (O.D.) values were detected at a wavelength of 450 nm.

### Inflammasome Activation

BMDMs were plated in 24-well plates at 5× 10^5^ cells per plate. After the cells adhered overnight, they were exposed for 4 h with DMEM containing LPS (50 ng/ml) or Pam3CSK4 (1 μg/ml). BMDMs were exposed to DHT for 1 h in Opti-MEM and then stimulated with nigericin (10 μM) for 30 min, ATP (5 mM) for 45 min, SiO_2_ (250 mg/ml) for 6 h, or *Salmonella* for 6 h. Besides, 1 μg/ml ultra-LPS, 2 μg/ml poly (I:C), or 2 μg/ml poly (dA:dT) were transfected with the StarFectII high-efficiency transfection reagent for 6 h.

### Western Blotting

The protein extraction of the cell culture supernatant was performed as described previously (Wang et al., 2019). The whole cell lysates were prepared with RIPA buffer. The samples were boiled at 105°C for 15 min and separated by 12% or 10% SDS-PAGE. Then, the gels were transferred to a nitrocellulose membrane by the wet transfer system. The membranes were sealed with 5% fat-free milk at room temperature for 1 h and incubated with primary and secondary antibodies in turn. The signals were analyzed using enhanced chemiluminescence reagents (Promega, Beijing, China).

### Enzyme-Linked Immunosorbent Assay

Mouse IL-1β (Dakewe, Beijing, China; R&D Systems, Minneapolis, MN, United States) and mouse TNF-α (Dakewe, Beijing, China) were used to measure the mouse serum, peritoneal lavage fluid, and cell supernatants following the manufacturer’s instructions.

### Caspase-1 Activity Assay

The activity of cleaved caspase-1 in the cell supernatant was measured by Caspase-Glo®1 Inflammasome Assay (Promega, Madison, Wisconsin, United States) following the manufacturer’s instructions.

### Flow Cytometry

The peritoneal lavage fluid was collected and centrifuged at 1,500 rpm at 4°C for 3 min. In consistence with the abovementioned centrifugation method, the cells were washed twice with PBS and discarded. Then, the cells were incubated with anti-F4/80 monoclonal antibody at 4°C in dark for 60 min. After incubation, the cells were washed with iced PBS 3 times. Finally, the cells were resuspended with 200 μl PBS and transferred to a flow tube for detection.

### Apoptosis-Associated Speck-Like Protein Containing a Card Oligomerization Assay

BMDMs were seeded in 12-well plates overnight. After inflammasome activation, the cell supernatant was removed, and the cells were lysed using Triton buffer [50 mM Tris-HCl (pH 7.5), 150 mM NaCl, 0.5% Triton X-100, and EDTA-free protease inhibitor cocktail]. The lysates were centrifuged at 6,000 g for 15 min. The supernatants detected the level of the NLRP3 inflammasome complex proteins by immunoblotting analysis, and the pellet fractions were washed with 500 μl PBS and resuspended in 200 μl PBS containing 2 mM DSS, and then cross-linked at 37°C for 30 min. After that, the pellets were centrifuged at 6,000 g for 15 min. Finally, the cell supernatant was removed.

### ROS Measurements

BMDMs were seeded in 100-mm cell culture dishes with 1 × 10^6^ cells/ml overnight. After that, the medium was replaced with fresh DMEM, and then primed with 50 ng/ml LPS for 4 h. Next, the cells were digested with trypsin (EDTA + trypsin) and stimulated with 10 μM nigericin for 30 min after being exposed to DHT for 1 h in Opti-MEM. Then, the cells were centrifuged at 5000 rpm for 5 min, and the supernatants were removed. After that, the cells were rinsed with HBSS and dyed with 4 μM MitoSOX for 20 min at 37°C. Flow cytometry was applied to detect the cells washed with HBSS.

### Intracellular K+ Measurement

BMDMs were plated in 24-well plates with a concentration of 1 × 10^6^ cells/ml overnight. The activation of the inflammasome has been described earlier. The medium was removed, and the cells were lysed with ultrapure HNO_3_. Next, the cell lysates were boiled at 100°C for 15 min. The content of intracellular K^+^ was measured by ICP-MS (inductively coupled plasma optical emission spectrometry).

### Intracellular Ca^2+^ Measurement

BMDMs were seeded at a concentration of 5 × 10^5^ cells/well in a 384-well plate. Then, DMEM containing LPS was used to replace the original medium. After 4 h, ATP was used to stimulate the cells for 45 min. DHT was used to treat or not treat the cells. ATP-induced Ca^2+^ fluxes were detected by the FLIPRT Tetra System (Molecular Devices, San Jose, CA, United States). The procedure has a complete introduction in the previous studies.

### Lipopolysaccharide-Induced Septic Shock Model

DHT was dissolved with 10% dimethyl sulfoxide and diluted with sterile saline. Before the experiment, 18–20 g C57BL/6 female mice were fed adaptively for a week. The septic shock model was established by intraperitoneal injection of LPS in mice. The vehicle control, DHT (40 mg/kg), or MCC950 (40 mg/kg) was injected intraperitoneally. LPS (20 mg/kg) was given 1 h later, and animal mortality was monitored regularly for three consecutive days. In another experiment, C57BL/6 female mice were randomly allocated to the following four groups: solvent control, LPS, MCC950 + LPS, and DHT + LPS group (*n* = 8). The administration condition was the same as that of the previous experiment, except that the serum samples and PBS peritoneal lavage fluid were adopted after 4 h. The cytokine levels were measured by ELISA, and the macrophages were analyzed by flow cytometry.

### Statistical Analysis

Statistical analysis was performed using GraphPad Prism 7 (GraphPad Software, San Diego, CA, United States) and Microsoft Excel. The data were expressed as the mean ± standard error of the mean (SEM). The unpaired Student’s *t*-test was used to analyze the significant differences between the two groups. The comparison of three or more groups used one-way ANOVA. The log-rank test was used to analyze the mice survival data. Statistical significance was indicated as *p < 0.05, **p < 0.01, and ***p < 0.001; ns: not significant.

## Results

### Dihydrotanshinone I Inhibits Nigericin and Adenosine Triphosphate–Induced Activation of the NLRP3 Inflammasome

**FIGURE 1:**
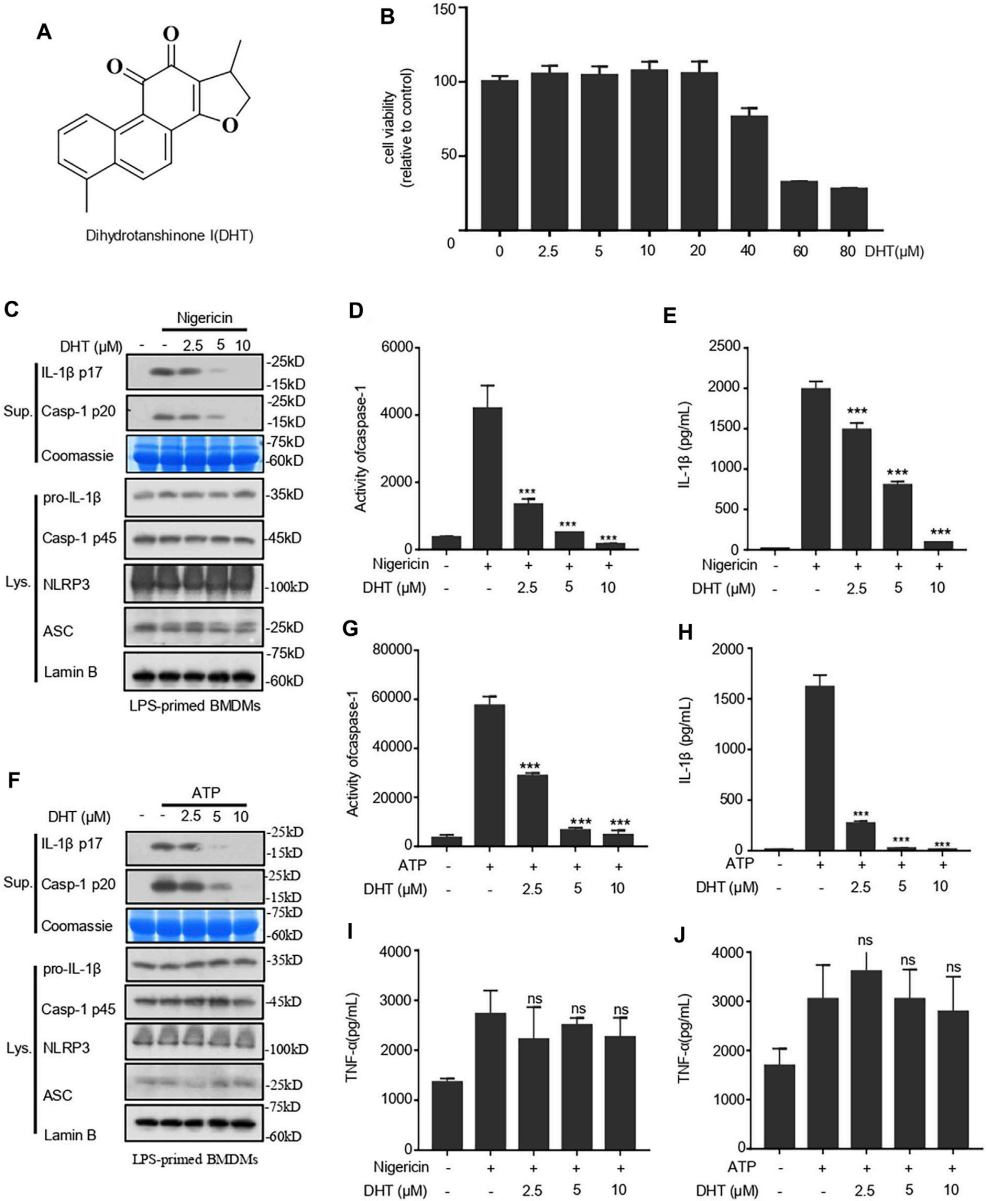
Dihydrotanshinone I (DHT) blocks NLRP3 inflammasome activation induced by nigericin and ATP in BMDMs. **(A)** Dihydrotanshinone I structure. **(B)** BMDMs were exposed to DHT (2.5–80 μM) for 24 h. **(C–E)** BMDMs were primed with LPS and then exposed to different concentrations (2.5, 5, or 10 μM) of dihydrotanshinone I, followed by the stimulation of nigericin for 0.5 h. Immunoblotting analysis of IL-1β (P17) and activated caspase-1 (P20) in cell supernatants (Sup.) are shown **(C)**. Caspase-1 activity **(D)** and IL-1β **(E)** secretion were measured. **(F–H)** BMDMs were primed with LPS and then treated with different concentrations (2.5, 5, or 10μM) of dihydrotanshinone I, followed by the stimulation of ATP for 1 h. Western blot analysis of matured IL-1β (P17) and activated caspase-1 (P20) in culture supernatants (Sup.) are shown **(F)**. Caspase-1 activity**(G)** and IL-1β **(H)** secretion are measured. **(I and J)** The secretion of TNF-a in the supernatant of cells treated as described in C **(I)** and F **(J)** were determined by ELISA. Coomassie blue staining was used as the loading control of the supernatant **(C, F)** and Lamin B was used as the lysate loading control **(C, F)**. Results are represented as mean ± SEM from three biological replicates. One-way ANOVA was used to analyze the data. *p < 0.05, **p < 0.01, ***p < 0.001, **NS:** not significant.

To examine whether dihydrotanshione I (DHT, Figure 1A) is influential in the activation of the NLRP3 inflammasome, we applied the CCK-8 kits to detect the cytotoxicity of DHT when different doses of DHT acted on macrophages derived from mouse bone marrow (BMDMs) for 24 h. It was observed that low concentrations of DHT did not damage the viability of BMDMs. DHT at concentrations below 20 μΜ is safe (Figure 1B).

Hence, the inhibition of DHT on the activity of caspase-1 and the secretion of IL-1β were measured at the concentration of 0–10 μΜ. We primed BMDMs with LPS and then treated them with DHT before being stimulated with nigericin. The immunoblotting results indicated that DHT treatment could suppress the cleavage of caspase-1 and the secretion of IL-1β (Figure 1C). Subsequently, the caspase-1 activity assay showed that DHT treatment and the activity of caspase-1 were dose-responsive (Figure 1D). Next, we measured the level of IL-1β by ELISA. ELISA results were consistent with those of Western blotting, indicating that DHT caused a dose-dependent inhibition of the activity of caspase-1 and the production of IL-1β (Figure 1E). Similarly, DHT dose dependently suppressed the level of ATP-induced caspase-1 and IL-1β (Figures 1F–H). However, DHT made no difference in the secretion of inflammasome-independent TNF-α (Figures 1I,J). Furthermore, the protein levels of NLRP3, ASC, pro–caspase-1, pro–IL-1β in the whole lysate of BMDMs were not affected by DHT (Figures 1C,F). Taken together, DHT efficiently blocks the activation of the NLRP3 inflammasome.

### Dihydrotanshinone I Specifically Inhibits the Activation of the NLRP3 Inflammasome but Does Not Affect AIM2 and NLRC4 Inflammasome Activation

**FIGURE 2:**
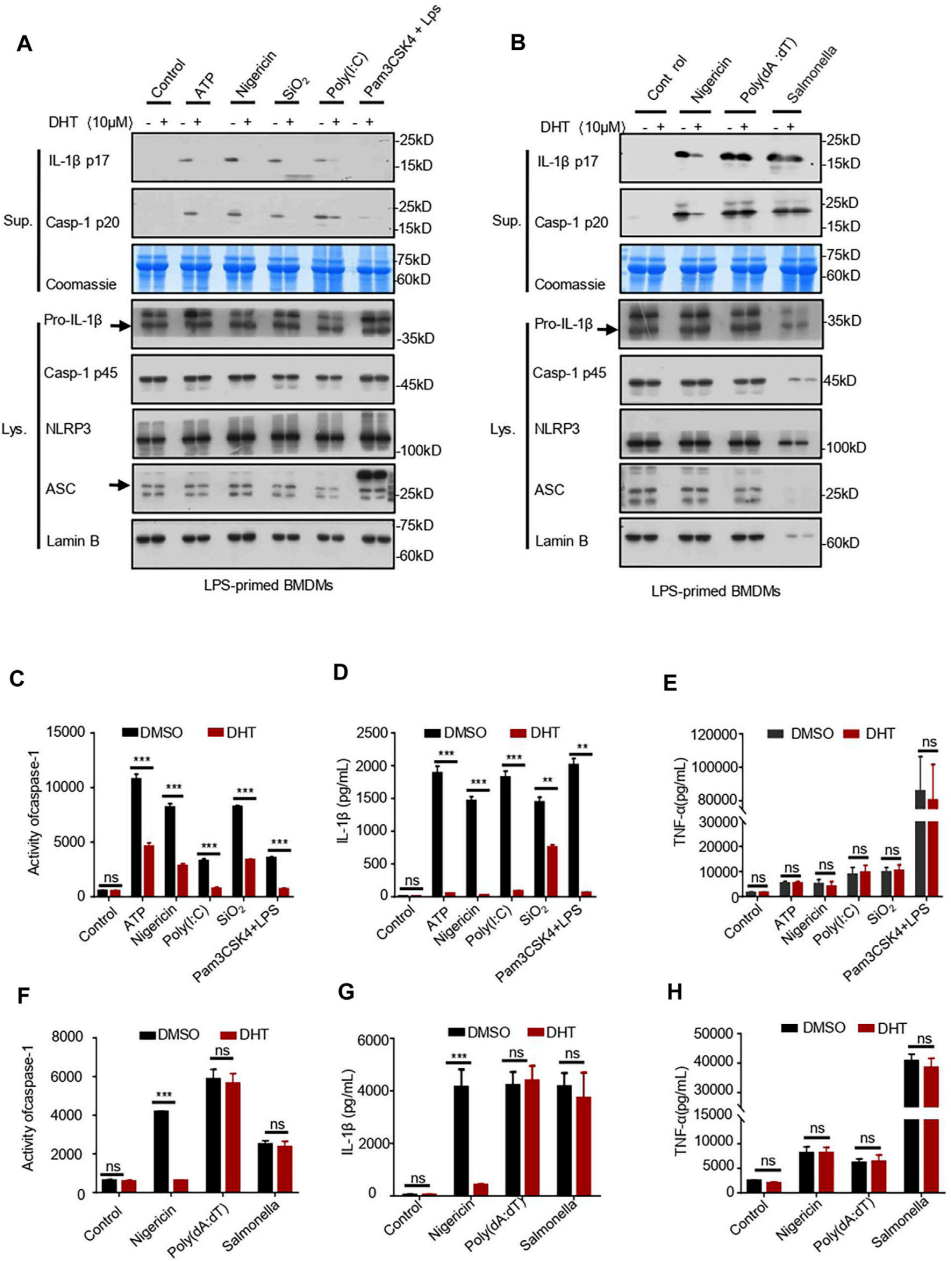
Dihydrotanshinone I specifically inhibits NLRP3 inflammasome activation. **(A)** LPS-primed BMDMs were treated with dihydrotanshinone I (10 μM) and then stimulated with ATP (45 min), nigericin (30 min), poly (I: C) (6 h), or SiO_2_(6 h), or Pam3CSK4-primed BMDMs were treated with dihydrotanshinone I (10 μM) and stimulated with LPS (6 h). Western blot analysis of IL-1β (p17) and caspase-1 (p20) in supernatants (Sup.) and pro–IL-1β and pro–caspase-1 in whole lysates (Lys.) of BMDMs are shown in **(A)**. **(B)** BMDMs were primed with LPS, exposed to DHT (10 μM), and then stimulated with nigericin, Poly (dA:dT) (6 h), and *Salmonella typhimurium* (6 h). Immunoblotting analysis of IL-1β (p17) and cleaved caspase-1 (p20) in culture supernatants (Sup.) and pro–IL-1β and pro–caspase-1 in whole lysates of BMDMs (Lys.). **(C–E)** Activity of caspase-1 **(C)**, secretion of IL-1β **(D)**, and production of TNF-α **(E)** in Sup. from samples described in **(A)**. **(F–H)** Activity of caspase-1 **(F)**, IL-1β **(G)**, and TNF-α **(H)** in Sup. from indicated samples in **(B)**. Coomassie blue staining was used as the supernatant loading control **(A–B)** and Lamin B as the lysate loading control **(A–B)**. Data are represented as mean ± SEM from three biological replicates. Statistics were analyzed by multiple t-tests. *p < 0.05, **p < 0.01, ***p < 0.001, **NS:** not significant.

To verify whether DHT can inhibit the NLRP3 inflammasome over a broad spectrum, we investigated the effects of DHT on the LPS-mediated canonical-activated pathway of SiO_2_ and poly (I: C). Our results indicated that DHT could inhibit SiO_2_- and poly (I: C)-induced production of IL-1β and activity of caspase-1 (Figures 2A,C,E). We also identified the role of DHT in the non-canonical activation of the NLRP3 inflammasome. Pam3CSK4-primed BMDMs were stimulated with LPS transfection after being exposed to DHT. The results showed that DHT reduced IL-1β secretion and caspase-1 cleavage (Figures 2. A,C,D). The data hint that DHT can restrain the canonical and non-canonical activation of the NLRP3 inflammasome in BMDMs.

Not only the NLRP3 inflammasome but also the AIM2 and NLRC4 inflammasomes may mediate IL-1β secretion and caspase-1 maturation. Subsequently, we tested whether DHT has a specific inhibitory effect on the activation of the NLRP3 inflammasome. We stimulated LPS-primed BMDMs with *Salmonella typhimurium* to see if DHT prevented the activation of the NLRC4 inflammasome (Samperio Ventayol et al., 2021). The results indicated that DHT did not influence the secretion of IL-1β and the maturation of caspase-1 during NLRC4 inflammasome activation in BMDMs (Figures 2B,F,G). It has been reported that double-stranded DNA can activate the AIM2 inflammasome (Li et al., 2021). LPS-primed BMDMs were exposed to DHT (10 μΜ) for 1 h and then transfected with poly (dA: dT) (Zhang C. et al., 2021). The effect of DHT on the AIM2 inflammasome was observed with no reduction in the secretion of IL-1β and cleavage of caspase-1 (Figures 2B,D,F). Meanwhile, DHT made no difference in the production of TNF-α (Figures 2E,H). The results supported DHT as a broad-spectrum inhibitor for the NLRP3 inflammasome, but it could not inhibit the activation of the AIM2 or NLRC4 inflammasome.

### Dihydrotanshinone I Inhibits ASC Oligomerization

**FIGURE 3:**
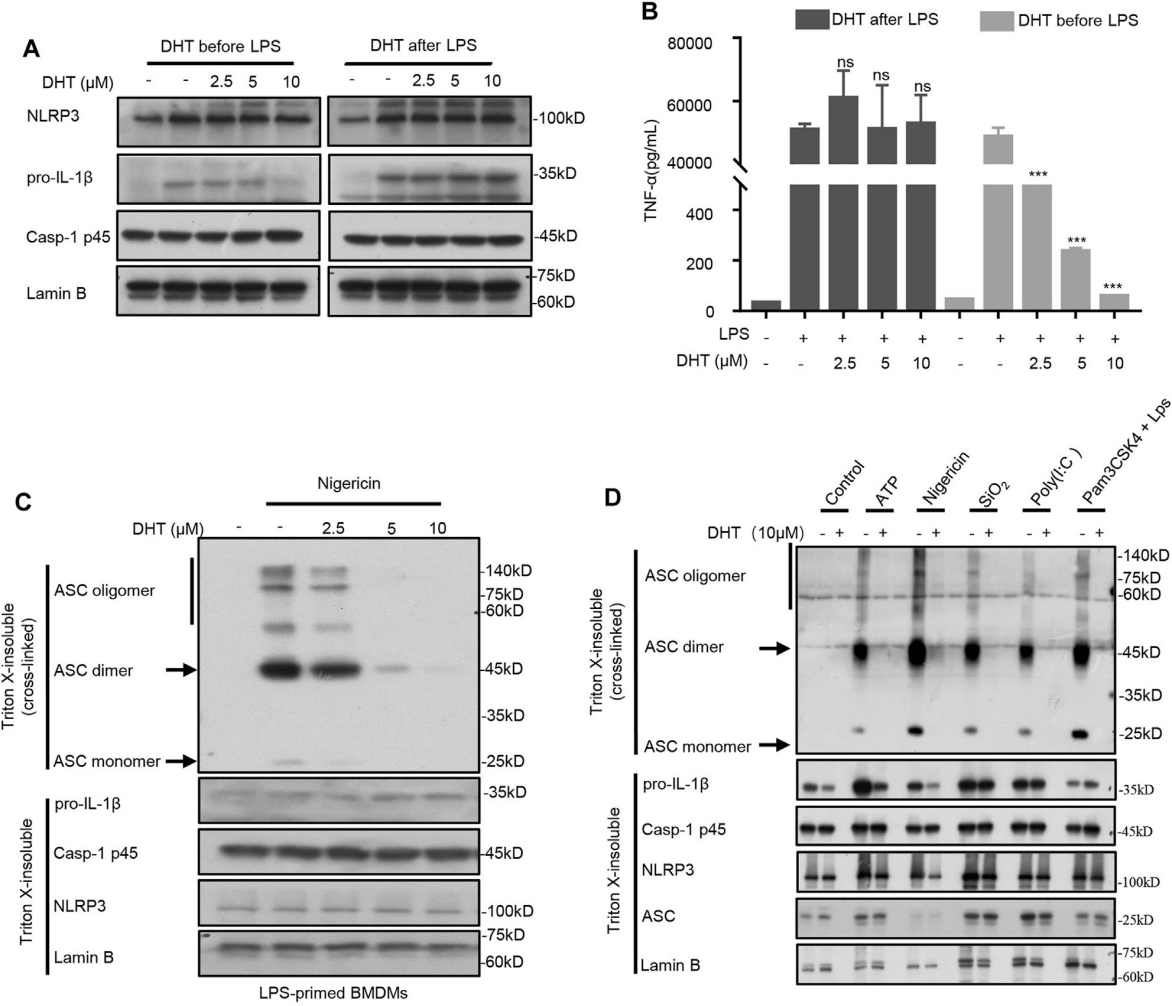
DHT inhibits ASC oligomerization. **(A)** Western blot analysis of proteins in lysates. from BMDMs with 4 h-LPS priming and then treated with or without different doses of DHT (2.5, 5, 10 μM) for 1 h, or BMDMs treated with varying doses of DHT (2.5, 5, 10 μM) for 1 h and then stimulated with LPS for 4 h. Lamin B was used as an internal control. **(B)** Production of TNF-α in Sup. from BMDMs as described in **(A)**. **(C, D)** BMDMs were pretreated with LPS and stimulated with nigericin, ATP, transfected LPS, SiO_2_, poly (I: C), or Pam3CSK4-primed BMDMs stimulated with LPS transfection after exposure to DHT. Western blotting analysis of ASC oligomerization in cell lysates.

Existing studies have shown that DHT blocks the TNF-*α*–induced NF-κB signaling pathway (Wang et al., 2015). Then, according to the method described earlier (Hou et al., 2020), we detected whether DHT affected the NF-κB–dependent expression of NLRP3 or pro–IL-1β to inhibit the activation of the NLRP3 inflammasome. We treated BMDMs with DHT for 1 h before being treated with 4 h-LPS. A high dose of DHT could downregulate the production of pro–IL-1β, NLRP3, and TNF-α (Figures 3A,B). Instead, when BMDMs were pretreated with LPS for 4 h and then followed by stimulation with or without DHT, the production of NLRP3, pro–IL-1β, and TNF-α remained unchanged (Figures 3A,B). In this condition, DHT showed inhibition of caspase-1 activity and IL-1β production (Figures 1A,F). Collectively, these data suggest that DHT, indeed, was effective in the activation stage of the NLRP3 inflammasome.

ASC oligomerization is a crucial stage during the activation of the inflammasome (Green et al., 2018). Then, we further explored whether DHT could block ASC oligomerization during the activation of the NLRP3 inflammasome. The LPS-primed BMDMs were stimulated by nigericin after being exposed to DHT, and then the cytoplasmic part of cell lysis was cross-linked. The ASC monomers and advanced complexes maintained a dose-dependent decline by DHT as observed by Western blotting (Figure 3C). Deeper results showed that DHT could inhibit the oligomerization of ASC mediated by multiple NLRP3 inflammasome stimuli, such as ATP, nigericin, SiO_2_, and poly (I: C) (Figure 3D). In comparison, DHT did not inhibit ASC oligomerization during *Salmonella typhimurium* and poly (dA: dT)-induced NLRC4 and AIM2 inflammasome activation (Supplementary Fig. 1). Thus, the results implied that DHT could inhibit ASC oligomerization when the NLRP3 inflammasome was activated.

### DHT Does Not Affect the Upstream Signals of NLRP3 Inflammasome Activation and the NLRP3–NEK7 Interaction

**FIGURE 4:**
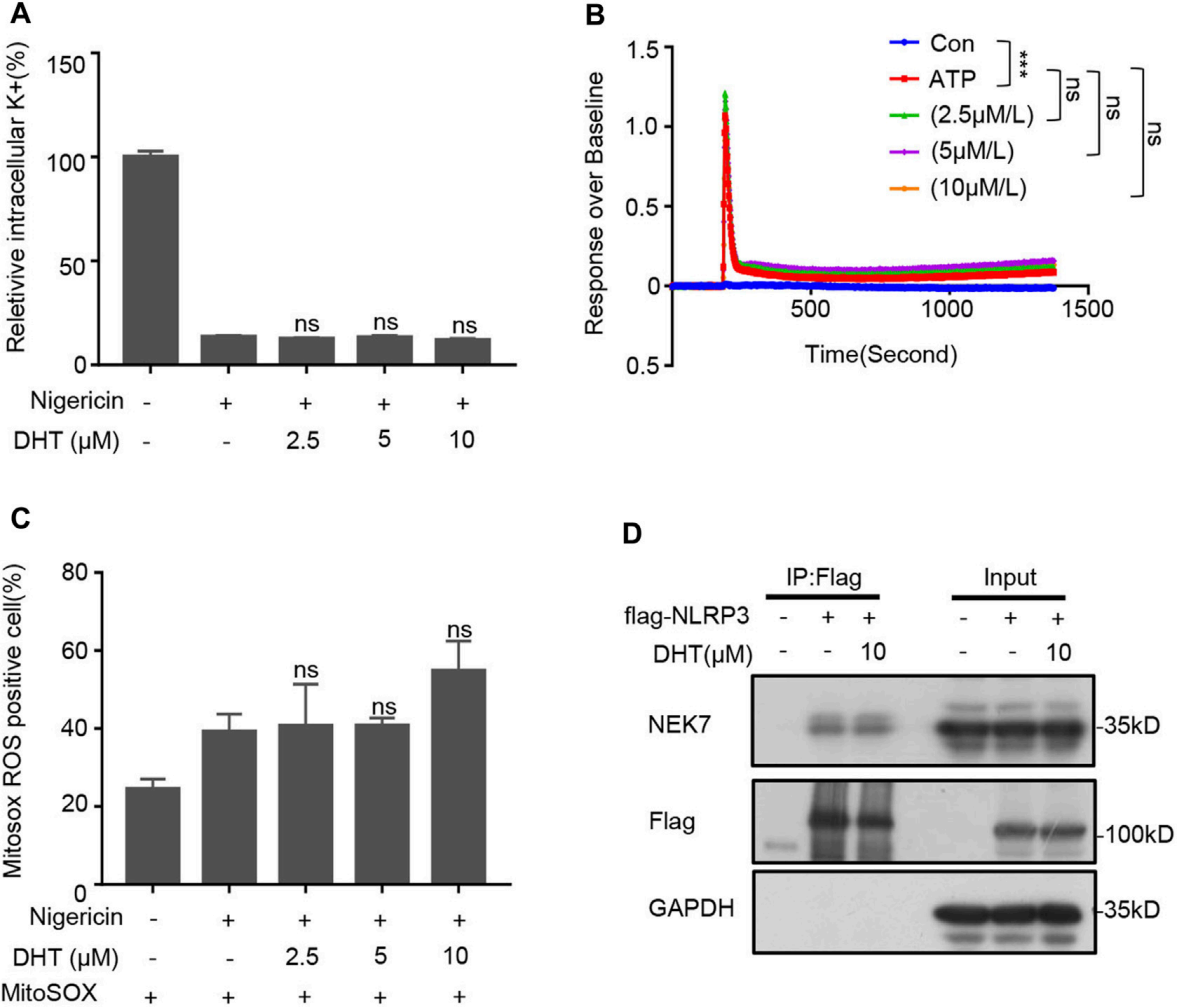
DHT has no effect on upstream signaling events of NLRP3 inflammasome activation and NLRP3–NEK7 interaction. **(A)** Qualification of potassium efflux in LPS-primed BMDMs exposed to various doses of DHT (2.5, 5, 10 μM) and then stimulated with nigericin. **(B)** ATP-induced Ca^2+^ flux in LPS-induced BMDMs treated with or without DHT was measured using a FLIPR Tetra system. **(C)** LPS-primed BMDMs treated with DHT and then stimulated with nigericin were detected by staining with MitoSox. The percentage of ROS-positive cells was obtained by flow cytometry. **(D)** HEK-293T cells were transfected with Flag-NLRP3, and DHT (10 μM) was added at 18 h post transfection. Immunoprecipitation was performed with anti-Flag affinity gel-agarose beads, followed by Western blot analysis. GAPDH was used as the lysate loading control. Data are shown as mean ± SEM from three biological replicates. One-way ANOVA was used to analyze the data. *p < 0.05, **p < 0.01, ***p < 0.001, NS: not significant.

The decrease of intracellular K^+^ concentration is recognized as a trigger for the activation of the NLRP3 inflammasome (Gong et al., 2018). Therefore, we tested if DHT prevented K^+^ efflux during NLRP3 activation. The results indicated that nigericin significantly reduced intracellular potassium levels, whereas DHT did not reverse such changes induced by nigericin (Figure 4A). Recent researches have clarified that Ca^2+^ signaling is a crucial signaling pathway in the activation of the NLRP3 inflammasome (Elliott and Sutterwala, 2015). Blocking Ca^2+^ signaling can inhibit the activation of the NLRP3 inflammasome but not AIM2 and NLRC4 inflammasomes (Elliott and Sutterwala, 2015). We observed that DHT did not affect ATP-induced calcium mobilization during NLRP3 inflammasome activation (Figure 4B). Moreover, oxidative stress also participates in the upstream of inflammasome activation, mitochondrial perturbations, and reactive oxygen species (mtROS) production which are essential for the activation of the NLRP3 inflammasome (He et al., 2016a; Verma et al., 2020). Thus, we investigated whether DHT influences the nigericin-induced ROS production. The emerging findings suggested that DHT treatment did not affect nigericin-induced mtROS production (Figure 4C).

NEK7 is an NLRP3-binding protein. The interaction between NEK7 and NLRP3, which plays a crucial role in regulating the assembly and activation of the NLRP3 inflammasome, is indispensable for activating the NLRP3 inflammasome (He et al., 2016b; Sharif et al., 2019). Therefore, we investigated if DHT was acting on the NEK7–NLRP3 interaction. 293T cell lines were transfected with Flag-NLRP3, and the immunoprecipitates were analyzed by Western blotting. Our results showed that DHT did not inhibit the binding of NEK7 to NLRP3 (Figure 4D). Taken together, DHT has no effect on the upstream signals of NLRP3 inflammasome activation, as well as the interaction between NLRP3 and NEK7.

### Dihydrotanshinone I Inhibits Inflammation *In Vivo* and Protects Against LPS-Induced Septic Shock *In Vivo*

**FIGURE 5:**
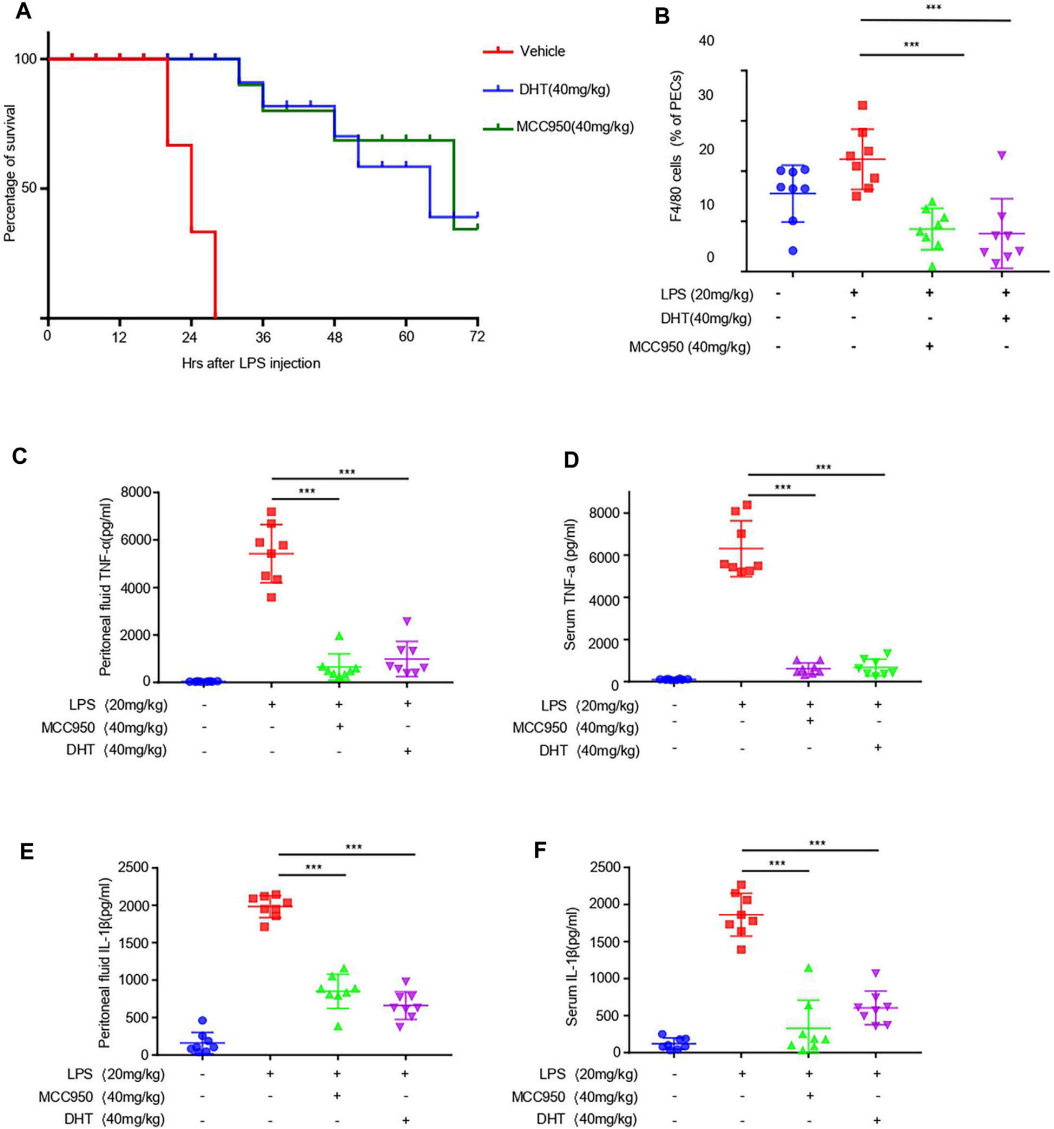
Dihydrotanshinone I reduces LPS-induced septic shock and inflammation *in vivo*. **(A)** Survival rate of septic shock in mice. After intraperitoneal injection of DHT (40 mg/kg) and LPS (20 mg/kg), the lethality of 6- to 8-week-old C57BL/6 female mice was monitored within 72 h (*n* = 10) (B–F) C57BL/6 female mice were given vehicle control, DHT (40 mg/kg), or MCC950 (40 mg/kg) for 1 h and then treated with LPS (20 mg/kg) for 4 h. Monocytes-macrophages (F4/80 + cells) **(B)**, TNF-α **(C)**, IL-1β **(E)** from serum and TNF-α **(D)**, and IL-1β **(F)** in the peritoneal lavage fluid were measured using flow cytometry and ELISA. Data are represented as the mean ± SEM. Statistics differences were analyzed using an unpaired Student’s *t*-test *p < 0.05, **p < 0.01, ***p < 0.001, **NS:** not significant.

To determine the role of DHT on the activation of the NLRP3 inflammasome *in vivo*, we selected the mouse model of LPS-induced NLRP3 inflammasome–dependent septic shock (Mao et al., 2013; Lee et al., 2017). We intraperitoneally injected DHT or MCC950 in mice, and LPS was injected 1 h later to monitor their survival. Our results showed that DHT significantly increased the survival rate of septic shock in mice injected by LPS (Figure 5A). On comparing the effects of DHT with MCC950, a selective inhibitor of the NLRP3 inflammasome, we observed that DHT had a similar protective effect on LPS-mediated death as MCC950 (Figure 5A). In addition, the mice were intraperitoneally treated with DHT or MCC950 for 1 h and then injected by LPS. The levels of IL-1β and TNF-α were evaluated 4 h later. The findings suggested that DHT had a similar effect as MCC950. DHT significantly reduced the levels of IL-1β and TNF-α in serum and peritoneal lavage fluid, and decreased the number of macrophages (Figures 5B–F). In conclusion, these results reveal that DHT treatment could block the activation of the NLRP3 inflammasome and improve NLRP3 inflammasome–mediated septic shock in mice.

## Discussion

*Salvia miltiorrhiza* is widely used in the treatment of inflammation. DHT is one of the main components of total tanshinones in *Salvia miltiorrhiza* and has an anti-inflammatory effect (Gao et al., 2018; Chen et al., 2019). However, the detailed mechanism of DHT in inflammatory diseases has not been fully elucidated. Current studies suggest that when the NLRP3 inflammasome is abnormally activated, it can cause severe inflammatory responses, leading to various human inflammatory diseases (Chen et al., 2009; Abderrazak et al., 2015; Louvrier et al., 2020). This study demonstrated that DHT acts as a specific inhibitor to block the canonical and non-canonical activation of the NLRP3 inflammasome but has no effect on the activation of AIM2 and NLRC4 inflammasomes. Evidence has emerged that DHT attenuates crystalline silica–induced lung inflammation by regulating the immune response and inhibiting STAT1/STAT3 (Zhang et al., 2019). DHT can promote amyloid-β accumulation clearance and decrease Tau phosphorylation by autophagy and the AMPK/mTOR pathway (Zhou et al., 2011; Bao et al., 2020). A previous study also showed that DHT could promote the formation of a negative feedback loop between the HuR level and TRIM21 expression under UV irradiation (Guha et al., 2020). In our study, we have shown that DHT could inhibit the activation of the NLRP3 inflammasome targeted by many types of factors, including nigericin, ATP, poly (I: C), or SiO_2_ (Figure 2). Thanks again for the reviewer’s suggestion, and we have added the discussions in our revised manuscript (page 13, line 326–334).

We also explored the mechanism of how DHT inhibits the activation of the NLRP3 inflammasome. Previous studies have reported that after the two steps of priming and activation are completed, the NLRP3 inflammasome can activate and subsequently secrete bioactive IL-1β (Jo et al., 2016; Duan et al., 2020). Reports showed that the activation of the NF-κB signaling pathway plays an important role on the expression of NLRP3 in the priming events (Yu et al., 2017; Wang et al., 2018). The ability of DHT to inhibit the activation of the NF-kB signaling pathway has long been proven (Wang et al., 2015). Consistent with the previous study, our results also demonstrated that DHT inhibited the inflammasome-independent production of pro–IL-1β and NLRP3 in DHT-pretreated BMDMs followed by LPS priming. In contrast, when BMDMs were treated with LPS prior to DHT stimulation, DHT has no effect on the expression of NLRP3 and pro–IL-1β induced by LPS. However, in this case, DHT can inhibit the caspase-1 maturation and the IL-1β secretion. These suggested that DHT could inhibit the priming phase of the NLRP3 inflammasome and play a part in the activation phase of the NLRP3 inflammasome.

mtROS production is a major upstream signaling regulator of NLRP3 inflammasome activation (Camilli et al., 2020). Our findings showed that DHT had no effect on the production of mtROS during the activation of the NLRP3 inflammasome. But, recent research studies claimed that DHT attenuated mtROS production in J774A.1 cells (Yue et al., 2021). In our results, the effect of DHT on mtROS production is not consistent with Hu et al.’s study, which may be caused by different cells and different experimental conditions. Potassium efflux (Di et al., 2018; Xu et al., 2020) and Ca^2+^ flux (Chae et al., 2015; Elliott and Sutterwala, 2015) are also the upstream signals of NLRP3 inflammasome activation. Our results also demonstrated that DHT had no inhibitory effect on calcium flux or potassium efflux. Thus, these results suggested that DHT does not influence the upstream signals of NLRP3 inflammasome activation. Therefore, we speculated whether DHT influences the activation of the NLRP3 inflammasome by targeting the assembly of the NLRP3 inflammasome. ASC oligomerization is an important assembly step in the activation of the NLRP3 inflammasome. These data indicated that DHT was provided with the solid repression of ASC oligomerization during the activation of the NLRP3 inflammasome. However, DHT did not affect NLRC4- and AIM2-dependent ASC oligomerization. Therefore, these data clarified that DHT may inhibit NLRP3 inflammasome assembly through the upstream events of ASC oligomerization, thereby inhibiting NLRP3 inflammasome activation. However, this unknown upstream event needs to be studied further.

Intraperitoneal injection of lipopolysaccharide induces sepsis (Ulevitch and Tobias, 1995; Liao et al., 2018), and the activation of the NLRP3 inflammasome plays a crucial role *in vivo*, accompanied by the production of IL-1β and the occurrence of inflammation (Lee et al., 2017; Xiong et al., 2020). In our study, the results showed that DHT could antagonize LPS-induced septic shock in mice and raise the survival rate. *In vivo* experiments showed that DHT inhibited the production of IL-1β and TNF-α in the serum and peritoneal lavage fluid of mice and inhibited the recruitment of macrophages in peritoneal lavage fluid, suggesting that DHT could inhibit the activation of the NLRP3 inflammasome *in vivo* and alleviate the disease mediated by the NLRP3 inflammasome. The therapeutic effect was almost the same as that of MCC950. In this study, only F4/80 was detected to label the macrophages. In future studies, the effect of DHT on inflammation *in vivo* can also be detected by detecting total cells and neutrophils in peritoneal lavage fluid. Moreover, previous studies have shown that DHT attenuates DSS-induced experimental ulcerative colitis in mice (Guo et al., 2018). It has been confirmed that DSS-induced ulcerative colitis is an NLRP3-dependent disease (Bauer et al., 2010; Ruiz et al., 2017). We speculate that DHT may ameliorate DSS-induced experimental ulcerative colitis by inhibiting the NLRP3 inflammasome. Therefore, our study proves DHT to be a potential agent for a position in the treatment for NLRP3 inflammasome–mediated diseases.

## Data Availability

The original contributions presented in the study are included in the article/Supplementary Material, further inquiries can be directed to the corresponding author/s.
