# Supplementary material for: Dihydrotanshinone I Specifically Inhibits NLRP3 Inflammasome Activation and Protects Against Septic Shock In Vivo
Source: Front Pharmacol. 2021 Oct 14;12:750815. doi: 10.3389/fphar.2021.750815 (PMC8552015; doi:10.3389/fphar.2021.750815)
Supplement: Supplementary file 1 [file Image1.pdf]

Dihydrotanshinone I specifically inhibits NLRP3 inflammasome activation. (A)LPS-primed BMDMs were treated with Dihydrotanshinone I (10 μM) and then stimulated with ATP (45 min), nigericin (30 min), poly (I: C) (6 h), SiO2(6 h), or Pam3CSK4-primed BMDMs were treated with Dihydrotanshinone I (10 μM) and stimulated with LPS (6 h). Western blot analysis of IL-1β (p17) and caspase-1 (p20) in supernatants (Sup.) and pro-IL-1β and pro-caspase-1 in whole lysates (Lys.) of BMDMs were shown in (A).

Supplemental Fig. 1. Dihydrotanshinone does not inhibit ASC oligomerization during NLRC4 and AIM2 inflammasomes activation

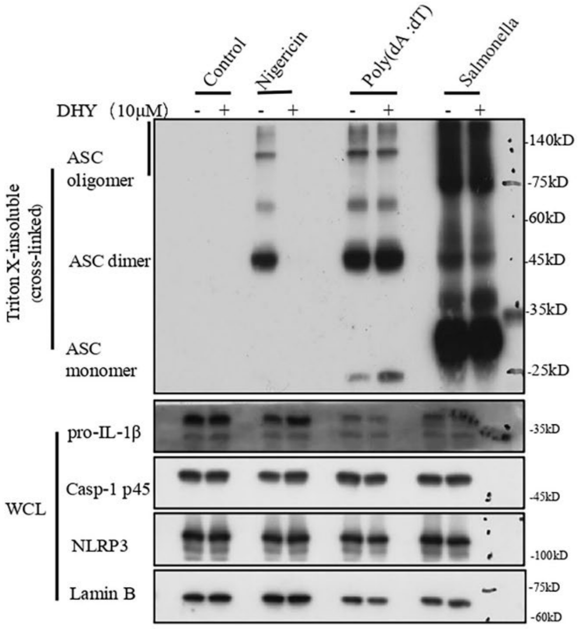

Supplementary figure1. Dihydrotanshinone I had no inhibition on NLRP3 inflammasome activation. LPS-primed BMDMs were treated with Dihydrotanshinone I (10 μM) and then stimulated with nigericin (30 min), poly (dA: dT) (6 h), Salmonella(6 h). Western blot analysis of IL-1β (p17) and caspase-1 (p20) in supernatants (Sup.) and pro-IL-1β and pro-caspase-1 in whole lysates (Lys.) of BMDMs were shown.
